# Supplementary material for: An inhibitory segment within G-patch activators tunes Prp43-ATPase activity during ribosome assembly
Source: Nat Commun. 2024 Nov 22;15:10150. doi: 10.1038/s41467-024-54584-5 (PMC11584650; doi:10.1038/s41467-024-54584-5)
Supplement: Supplementary file 1 — Supplementary Information [file 41467_2024_54584_MOESM1_ESM.pdf]

Supplementary Information

An inhibitory segment within G-patch activators tunes Prp43-ATPase activity during ribosome assembly

Daniela Portugal-Calisto, Alexander Gregor Geiger, Julius Rabl, Oscar Vadas, Michaela Oborská-Oplová, Jarosław Mazur, Federica Richina, Purnima Klingauf-Nerurkar, Erich Michel, Alexander Leitner, Daniel Boehringer & Vikram Govind Panse*

*Corresponding author. e-mail: vpanse@imm.uzh.ch

Table of Contents

1. Supplementary Tables 1 to 3 (p. 2 – 6)
2. Supplementary Figures 1 to 6 (p. 7 – 16)
3. Uncropped SDS-PAGE gels (p. 17)
4. Supplementary References (p. 18)

Supplementary Table 1. Cryo-EM data collection, refinement, and validation statistics

	dataset 1	dataset 2
**Data collection and processing**		
Magnification	105 000	130 000
Voltage (kV)	300	300
Electron exposure (e–/Å^2)	85	80
Defocus range (μm)	-1 to -3	-1 to -3
Pixel size (Å)	0.84	0.66
Symmetry imposed	C1	
Initial particle images (no.)	3 087 725	
Final particle images (no.)	258 878	
Map resolution (Å)	3.3	
FSC threshold	0.143	
Map resolution range (Å)	2.9 - 7.5	
**Refinement**		
Initial model used (PDB code)	AlphaFold model	
Model resolution (Å)	3.5	
FSC threshold	0.5	
Map sharpening B factor (Å^2)	-159.5	
Model composition		
Non-hydrogen atoms	5 504	
Protein residues	680	
Ligands	1	
B factors (Å^2)		
Protein	8.04/134.07/64.70	
Ligand	26.49/55.65/47.51	
R.m.s. deviations		
Bond lengths (Å)	0.003	
Bond angles (°)	0.517	
Validation		
MolProbity score	1.28	
Clashscore	3.19	
Poor rotamers (%)	0.33	
Ramachandran plot		
Favored (%)	97.03	
Allowed (%)	2.97	
Disallowed (%)	0	

Supplementary Table 2. Yeast strains used in this study

Strain	Genotype	Origin
BY4741	*MATa ura3 his3 leu2 met15 TRP1*	Euroscarf
NMY32	*MATa trp1 leu2 (lexAop)8-ADE2 LYS2::(lexAop)4-HIS3 URA3::(lexAop)8-lacZ GAL4*	Dualsystems Biotech AG
Tma23-TAP	*MATa ura3 leu2 TRP1 TMA23-TAP::HIS3MX*	Open biosystems
Pxr1-TAP	*MATa ura3 leu2 TRP1 PXR1-TAP::HIS3MX*	Open biosystems
Prp43-TAP	*MATa ura3 leu2 TRP1 PRP43-TAP::HIS3MX*	Open biosystems
P_{GAL1}-TMA23	*MATa ura3 his3 leu2 met15 TRP1* P_{GAL1}*-TMA23::natNT2*	This study
P_{GAL1}-PXR1	*MATa ura3 his3 leu2 met15 TRP1* P_{GAL1}*-PXR1::natNT2*	This study
bud20Δ	*MATa his3 leu2 met15 ura3 BUD20::KANMX*	Altvater *et al.*, 2012[1]
yrb2Δ	*MATa his3 leu2 met15 ura3 YRB2::KANMX*	Altvater *et al.*, 2014[2]
TMA23-GFP	*MATa ura3 leu2 met15 TRP1 TMA23-GFP::HIS3MX*	Open biosystems
PXR1-GFP	*MATa ura3 leu2 met15 TRP1 PXR1-GFP::HIS3MX*	Open biosystems
TMA23-GFP GAR1-mCherry	*MATa ura3 leu2 met15 TRP1 TMA23-GFP::HIS3MX GAR1- mCherry::kanMX6*	This study
PXR1-GFP GAR1-mCherry	*MATa ura3 leu2 met15 TRP1 PXR1-GFP::HIS3MX GAR1-mCherry::kanMX6*	This study

Supplementary Table 3. Plasmids used in this study

Plasmid	Relevant markers	Source
pRS315-*TMA23*	*TMA23 CEN LEU2 AMP*	This study
pRS315-*TMA23*^{L7E}	*TMA23 L7E CEN LEU2 AMP*	This study
pRS315-*PXR1*	*PXR1 CEN LEU2 AMP*	This study
pRS315-*PXR1*^{L33E}	*PXR1 L33E CEN LEU2 AMP*	This study
pRS315-*PXR1*^{F126A}	*PXR1 F126A CEN LEU2 AMP*	This study
pRS315-*PXR1*^{GM}-*TMA23*^{D}	*PXR1*^{GM} *(1-137)-TMA23*^{D} *(111-211) CEN LEU2 AMP*	This study
pRS315-*PXR1*$^{GM\text{-}F126A}$-*TMA23*^{D}	*PXR1*^{GM} *(1-137)F126A-TMA23*^{D} *(111-211) CEN LEU2 AMP*	This study
pRS315-*TMA23*^{GM}-*PXR1*^{D}	*TMA23*^{GM} *(1-110)-PXR1*^{D} *(138-271) CEN LEU2 AMP*	This study
pRS315-*TMA23*$^{GM\text{-}F96A}$-*PXR1*^{D}	*TMA23*^{GM} *(1-110) F96A-PXR1*^{D} *(138-271) CEN LEU2 AMP*	This study

pRS315-*Pxr1*^{GM}-*Cov2*^{C}	*PXR1*^{GM} *(1-137)-COV2*^{C} *(250-364) CEN LEU2 AMP*	This study
pRS315-*Pxr1*$^{GM-F126A}$-*Cov2*^{C}	*PXR1*^{GM} *(1-137) F126A-COV2*^{C} *(250-364) CEN LEU2 AMP*	This study
pRS315-*TMA23*^{GM}-*Cov2*^{C}	*TMA23*^{GM} *(1-110)-COV2*^{C} *(250-364) CEN LEU2 AMP*	This study
pRS315-*TMA23*$^{GM-F96A}$-*Cov2*^{C}	*TMA23*^{GM} *(1-110) F96A-COV2*^{C} *(250-364) CEN LEU2 AMP*	This study
pRS315-*uL18-GFP*	*RPL5-GFP CEN LEU2 AMP*	Altvater *et al.*, 2012[1]
pRS315-*uS5-eGFP*	*RPS2-eGFP CEN LEU2 AMP*	Altvater *et al.*, 2014[2]
pRS313-*TMA23*	*TMA23 CEN HIS3 AMP*	This study
pRS313-*TMA23*^{F96A}	*TMA23 F96A CEN HIS3 AMP*	This study
pLexA-*PRP43*	*LexA-PRP43 2μ TRP1 AMP*	This study
pLexA-*PXR1*	*LexA-PXR1 2μ TRP1 AMP*	This study
pLexA-*PXR1*^{GM}	*LexA-PXR1*^{GM} *(1-137) 2μ TRP1 AMP*	This study
pLexA-*PXR1*^{GM} *L33E*	*LexA-PXR1*^{GM} *(1-137) L33E 2μ TRP1 AMP*	This study
pLexA-*PXR1*^{GM} *W38A*	*LexA-PXR1*^{GM} *(1-137) W38A 2μ TRP1 AMP*	This study
pLexA-*PXR1*^{GM} *L44E*	*LexA-PXR1*^{GM} *(1-137) L44E 2μ TRP1 AMP*	This study
pLexA-*PXR1*^{GM} *G66E*	*LexA-PXR1*^{GM} *(1-137) G66E 2μ TRP1 AMP*	This study
pLexA-*PXR1*^{GM} *L67E*	*LexA-PXR1*^{GM} *(1-137) L67E 2μ TRP1 AMP*	This study
pLexA-*PXR1*^{G}	*LexA-PXR1*^{G} *(1-79) 2μ TRP1 AMP*	This study
pLexA-*PXR1*^{M}	*LexA-PXR1*^{M} *(72-137) 2μ TRP1 AMP*	This study
pLexA-*PXR1*$^{M-F126A}$	*LexA-PXR1*^{M} *(72-137) F126A 2μ TRP1 AMP*	This study
pLexA-*PXR1*^{D}	*LexA-PXR1*^{D} *(137-271) 2μ TRP1 AMP*	This study
pLexA-*TMA23*	*LexA-TMA23 (1-211) 2μ TRP1 AMP*	This study
pLexA-*TMA23*^{D}	*LexA-TMA23*^{D} *(110-211) 2μ TRP1 AMP*	This study
pLexA-*LAMINC*	*LexA-LAMINC 2μ TRP1 KAN*	Dual Systems
pACT2.2-*TMA23*	*GAL4 AD-TMA23 2μ LEU2 AMP*	This study
pACT2.2-*TMA23*^{GM}	*GAL4 AD-TMA23*^{GM} *(1-110) 2μ LEU2 AMP*	This study
pACT2.2-*TMA23*^{GM} *L7E*	*GAL4 AD-TMA23*^{GM} *(1-110) L7E 2μ LEU2 AMP*	This study
pACT2.2-*TMA23*^{GM} *W12A*	*GAL4 AD-TMA23*^{GM} *(1-110) W12A 2μ LEU2 AMP*	This study

pACT2.2-*TMA23*^{GM} *F18E*	*GAL4 AD-TMA23*^{GM} *(1-110) F18E 2μ LEU2 AMP*	This study
pACT2.2-*TMA23*^{GM} *G37E*	*GAL4 AD-TMA23*^{GM} *(1-110) G37E 2μ LEU2 AMP*	This study
pACT2.2-*TMA23*^{GM} *L38E*	*GAL4 AD-TMA23*^{GM} *(1-110) L38E 2μ LEU2 AMP*	This study
pACT2.2-*TMA23*^{G}	*GAL4 AD-TMA23*^{G} *(1-53) 2μ LEU2 AMP*	This study
pACT2.2-*TMA23*^{M}	*GAL4 AD-TMA23*^{M} *(43-110) 2μ LEU2 AMP*	This study
pACT2.2-*TMA23*$^{M-F96A}$	*GAL4 AD-TMA23*^{M} *(43-110) F96A 2μ LEU2 AMP*	This study
pACT2.2-*TMA23*^{D}	*GAL4 AD-TMA23*^{D} *(110-211) 2μ LEU2 AMP*	This study
pACT2.2-*TMA23*^{GM}*-Pxr1*^{D}	*GAL4 AD-TMA23*^{GM} *(1-110) 2μ LEU2 AMP*	This study
pACT2.2-*TMA23*$^{GM-F96A}$*-Pxr1*^{D}	*GAL4 AD-TMA23*^{GM} *(1-110) F96A 2μ LEU2 AMP*	This study
pACT2.2-*PXR1*	*GAL4 AD-PXR1 2μ LEU2 AMP*	This study
pACT2.2-*PXR1*^{GM}	*GAL4 AD-PXR1*^{GM} *(1-137) 2μ LEU2 AMP*	This study
pACT2.2-*PXR1*^{D}	*GAL4 AD-PXR1*^{D} *(137-271) 2μ LEU2 AMP*	This study
pACT-*PRP43*	*GAL4 AD-PRP43 2μ LEU2 AMP*	This study
pACT-*LargeT*	*GAL4 AD-SV40 largeT antigen 2μ LEU2 AMP*	Dual Systems
pET47b-*PRP43*	*HIS*$_{6}$*-PRP43 KAN*	This study
pET47b-*PRP43-T*^{G}	*HIS*$_{6}$*-PRP43-linker-TMA23*^{G} *(1-53) KAN* *Linker:* GGGSGGGSGGGSGSPW	This study
pET47b-*PRP43-P*^{G}	*HIS*$_{6}$*-PRP43-linker-Pxr1*^{G} *(1-79) KAN* *Linker:* GGGSGGGSGSPW	This study
pGEX-6P-1	*GST AMP*	Demmel *et al.*, 2008[3]
pGEX-6P-1-*TMA23*^{GM}	*GST-TMA23*^{GM} *(1-111) AMP*	This study
pGEX-6P-1-*TMA23*^{GM} *L7E*	*GST-TMA23*^{GM} *(1-111) L7E AMP*	This study
pGEX-6P-1-*TMA23*^{GM} *W12A*	*GST-TMA23*^{GM} *(1-111) W12A AMP*	This study
pGEX-6P-1-*TMA23*^{GM} *F18E*	*GST-TMA23*^{GM} *(1-111) F18E AMP*	This study
pGEX-6P-1-*TMA23*^{GM} *G37E*	*GST-TMA23*^{GM} *(1-111) G37E AMP*	This study
pGEX-6P-1-*TMA23*^{GM} *L38E*	*GST-TMA23*^{GM} *(1-111) L38E AMP*	This study
pGEX-6P-1-*TMA23*^{G}	*GST-TMA23*^{G} *(1-53) AMP*	This study
pGEX-6P-1-*TMA23*^{M}	*GST-TMA23*^{M} *(43-111) AMP*	This study
pGEX-6P-1-*TMA23*^{M44}	*GST-TMA23*^{M} *(68-111) AMP*	This study

pGEX-6P-1-*TMA23^{M} F96A*	*GST-TMA23^{M} (43-111) F96A AMP*	This study
pGEX-6P-1-*TMA23^{D}*	*GST-TMA23^{D} (111-211) AMP*	This study
pGEX-6P-1-*PXR1^{GM}*	*GST-PXR1^{GM} (1-137) AMP*	This study
pGEX-6P-1-*PXR1^{GM} L33E*	*GST-PXR1^{GM} (1-137) L33E AMP*	This study
pGEX-6P-1- *PXR1^{GM} W38A*	*GST-PXR1^{GM} (1-137) W38A AMP*	This study
pGEX-6P-1- *PXR1^{GM} L44E*	*GST-PXR1^{GM} (1-137) L44E AMP*	This study
pGEX-6P-1- *PXR1^{GM} G66E*	*GST-PXR1^{GM} (1-137) G66E AMP*	This study
pGEX-6P-1- *PXR1^{GM} L67E*	*GST-PXR1^{GM} (1-137) L67E AMP*	This study
pGEX-6P-1-*PXR1^{G}*	*GST-PXR1^{G} (1-79) AMP*	This study
pGEX-6P-1-*PXR1^{M}*	*GST-PXR1^{M} (72-137) AMP*	This study
pGEX-6P-1-*PXR1^{M14}*	*GST-PXR1^{M} (124-137) AMP*	This study
pGEX-6P-1-*PXR1^{M} F126A*	*GST-PXR1^{M} (72-137) F126A AMP*	This study
pGEX-6P-1-*PXR1^{D}*	*GST-PXR1^{D} (137-271) AMP*	This study
pGEX-6P-1-*PFA1^{G}*	*GST-PFA1^{G} (710-767) AMP*	This study

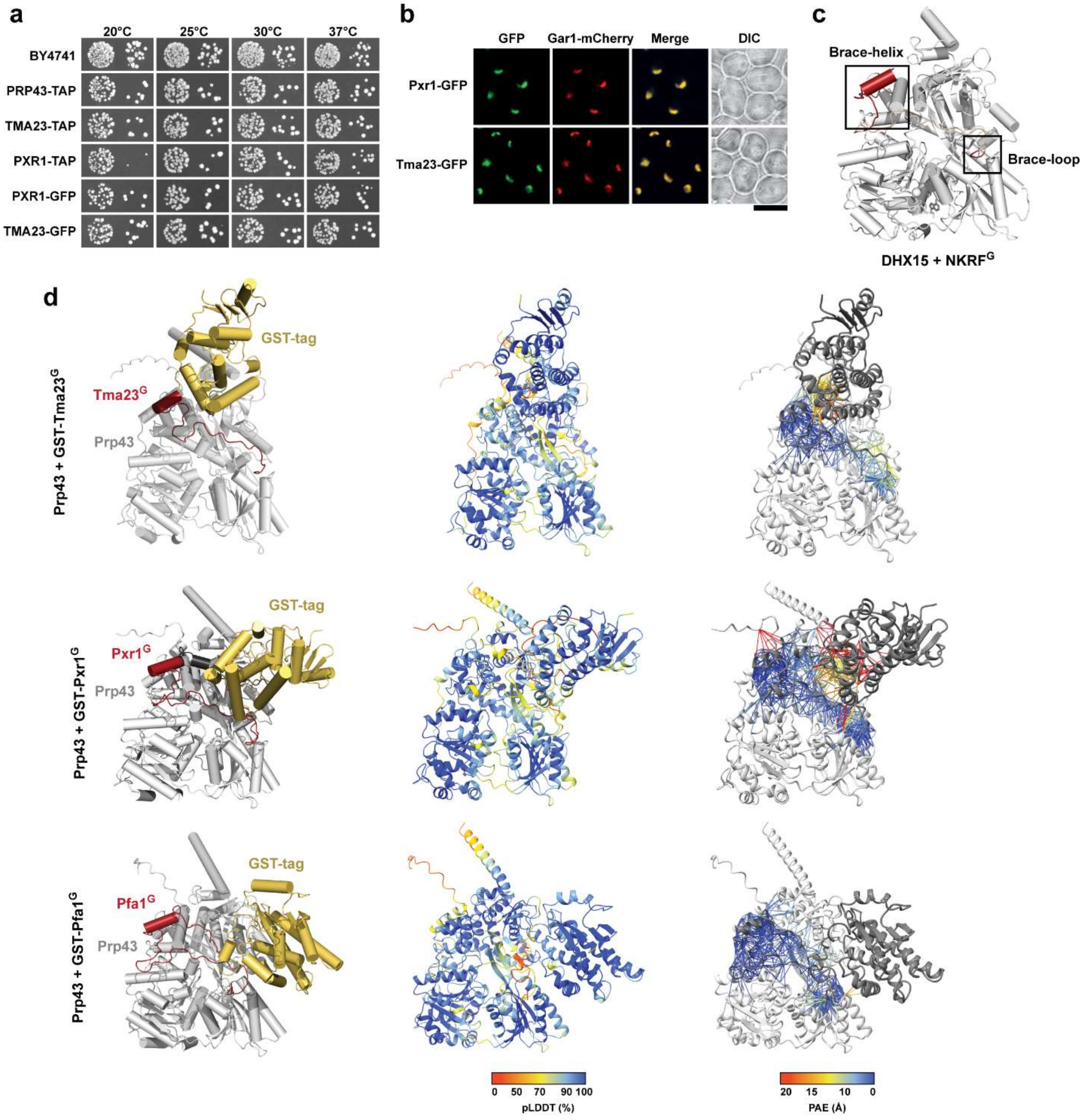

Supplementary Figure 1. (legend on next page)

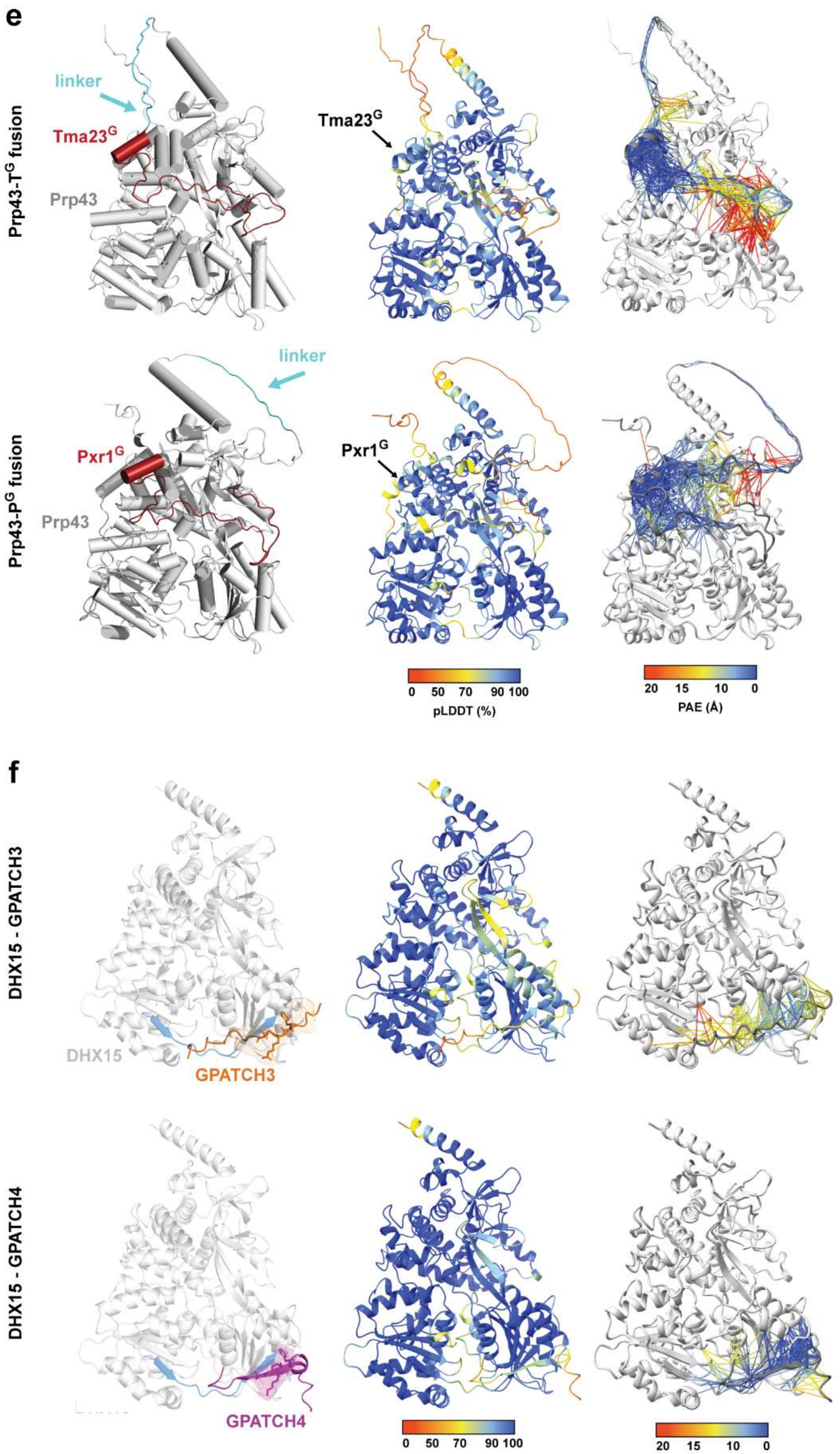

Supplementary Figure 1. C-terminal tagging of Prp43, Pxr1 and Tma23 strains does not compromise protein function. a, Different strains were spotted in 10-fold serial dilutions on YPD plates and incubated

at the indicated temperatures for 2 – 5 days. BY4741 cells were used as control. **b,** Cellular location of C-terminal fusions of Pxr1 and Tma23 to GFP were localized by fluorescence microscopy. Gar1-mCherry served as a nucleolar marker. Scale bar = 5 µm. **c,** Cartoon representation of DHX15:NKRF complex shows how G-patches bind to the RNA helicase (PDB 6SH6)[4]. The G-patch is depicted in red and the position of the brace-helix and the brace-loop within $NKRF^G$ is highlighted by black squares. Helices are shown as cylinders. **d,** AlphaFold2-derived models of Prp43 (grey) in complex with different G-patch segments (red) fused to N-terminal GST tags (yellow). The complexes are shown with cartoon representation and helices are depicted as cylinders. **e,** AlphaFold2-derived models of Prp43-T^G and Prp43-P^G fusion constructs. The proteins were fused by a glycine-serine linker displayed in blue. **f,** AlphaFold2-derived models of DHX15 (light grey) with putative I-patches of known human G-patch DHX15 cofactors (orange and magenta) show the same binding site as for the yeast Prp43 (highlighted in blue). **(d-f),** Predicted local distance difference test (pLDDT) and predicted aligned error (PAE) scores for the indicated AlphaFold2-predicted complexes. The pseudobonds indicating the PAE between pairs of residues were set for 8 Å. Structures were analyzed using ChimeraX (version 1.7.1, UCSF Chimera, National Institutes of Health, CA, USA)[5].

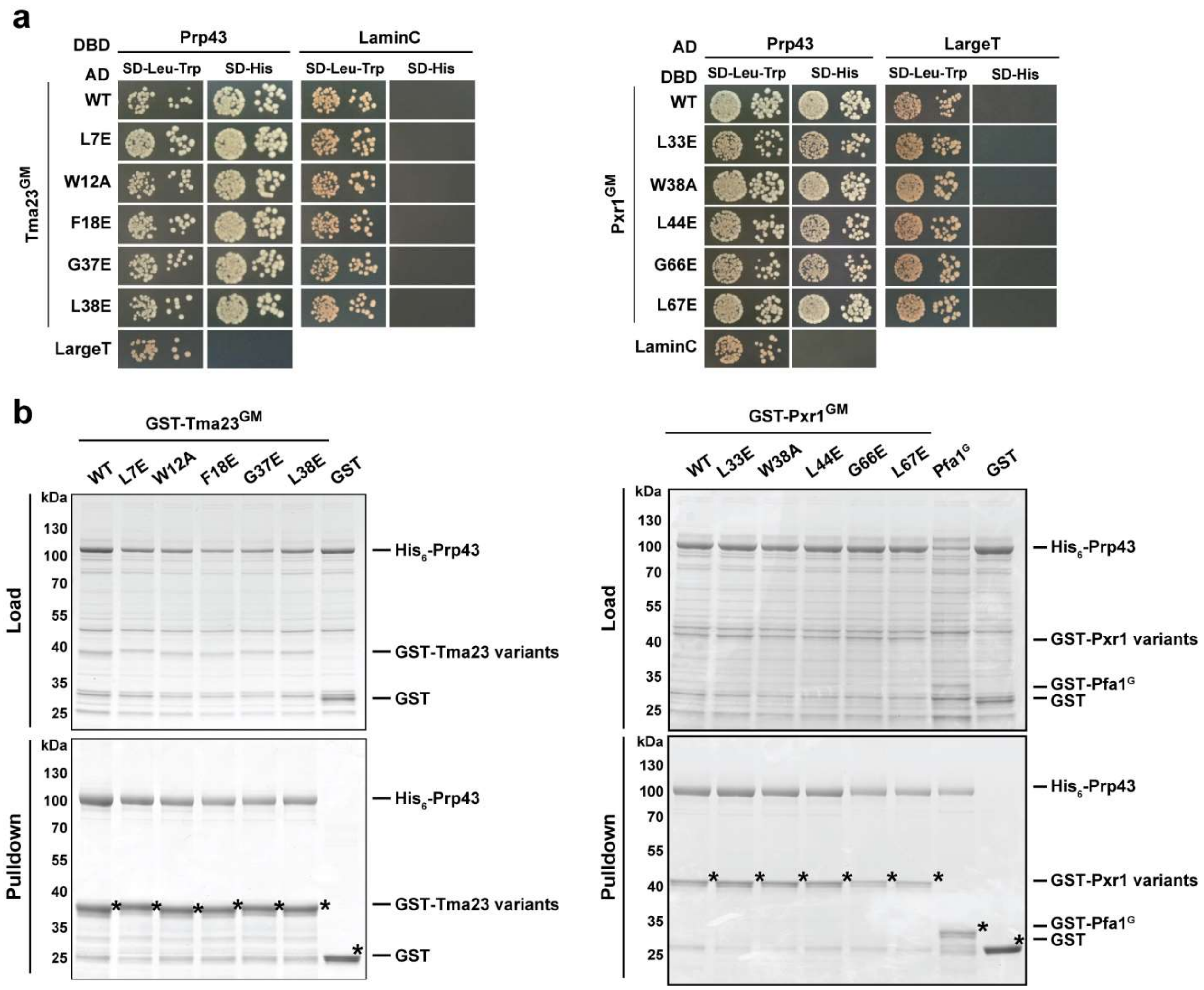

Supplementary Figure 2. Point mutants within Tma23^{G} and Pxr1^{G} do not abolish binding to Prp43. **a,** Yeast NMY32 strain transformed with plasmids encoding for the indicated *LexA* DNA-binding domain (DBD) and *GAL4* activation domain (AD) fusion proteins was spotted in 10-fold serial dilutions on the indicated medium and incubated at 30ºC for 2 – 4 days. LaminC and LargeT were used as negative controls. **b,** Recombinant His$_6$-Prp43 was co-expressed in *E. coli* with the indicated GST-fused Tma23^{G} and Pxr1^{G} wild type or mutant constructs, with Pfa1^{G}, or GST and affinity-purified using Glutathione Sepharose 4 Fast Flow beads (Cytiva). Proteins were eluted by heating the samples to 70ºC, separated on a SurePAGE 4-20% BIS-TRIS gradient gel by SDS-PAGE, and visualized by Coomassie Blue staining. Pfa1^{G} and GST were used as positive and negative controls, respectively. Asterisks indicate the baits. kDa refers to protein molecular weight.

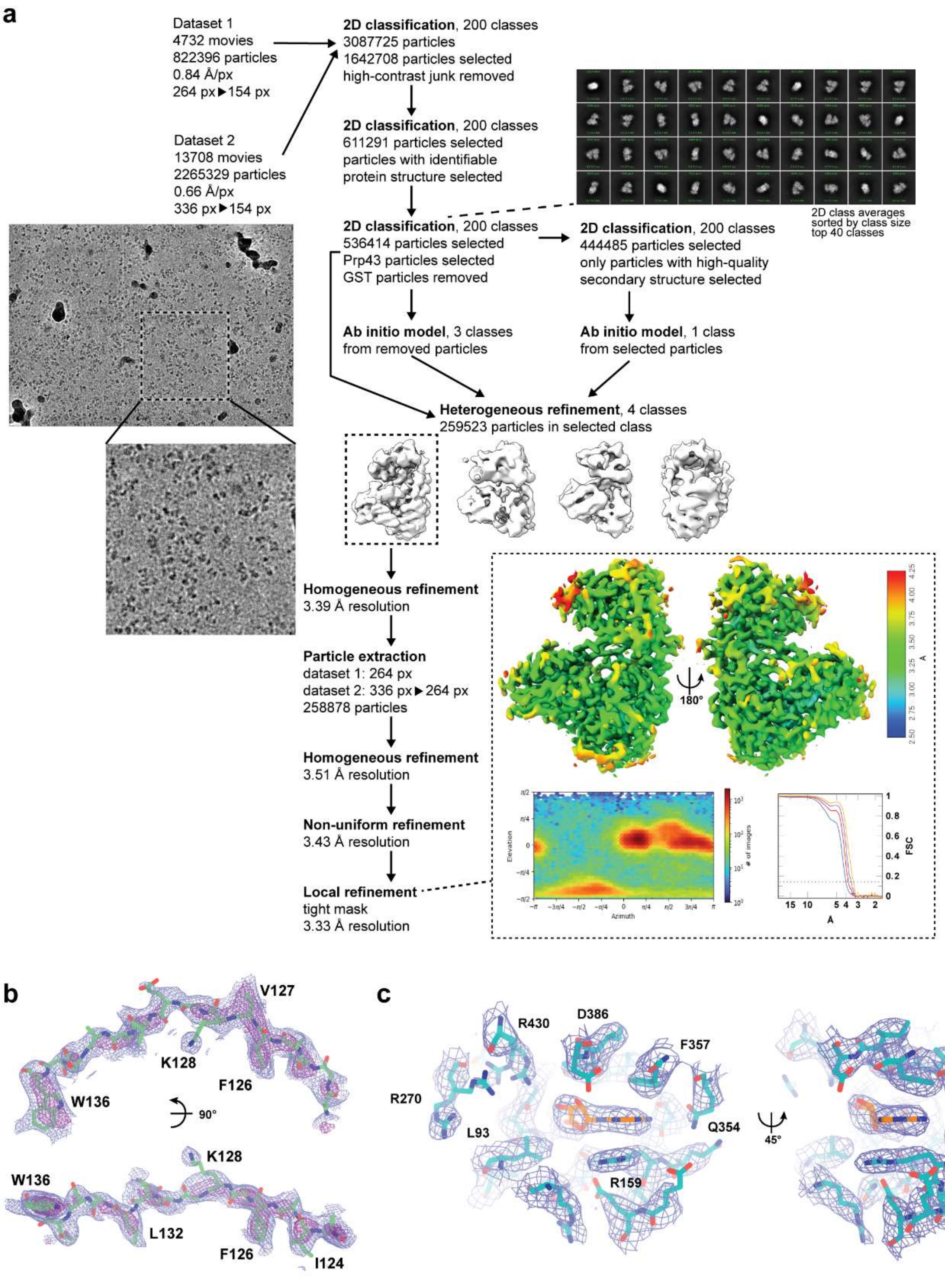

Supplementary Figure 3. Single particle cryo-EM processing workflow. a, Schematic representation of the cryo-EM image processing workflow of the Prp43-Pxr1^{GM} complex. The resolution map, the orientation distribution plot and the FSC plot (blue: unmasked, red: circular mask, purple: loose mask, orange: tight mask) of the final reconstruction are shown in the inset. A representative micrograph is shown. **b,** The experimental cryo-EM density around Pxr1 is shown as blue (sigma=4.0) and purple (sigma=10.0)

mesh. The refined atomic model of Pxr1 is shown as green stick model. **c,** Experimental density at the active site shown in mesh representation, with threshold 8 sigma.

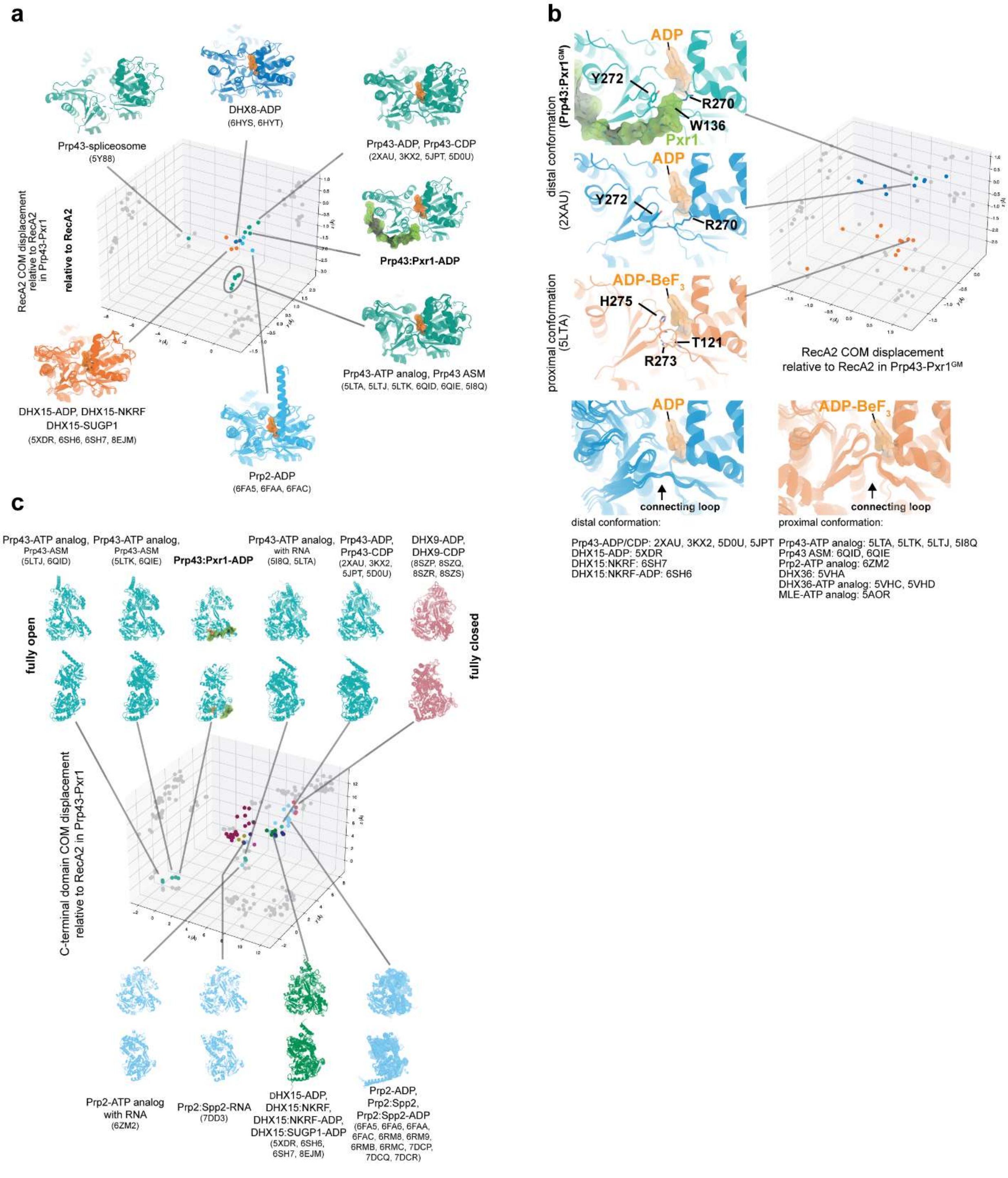

Supplementary Figure 4. Comparative analysis of the structural conformation of Prp43-Pxr1^{GM} complex with reported structures of Prp43 and other DEAH/RHA RNA helicases. a, Comparison of center of mass 3D-displacement (in Å) of the RecA2 domain concerning its position in Prp43:Pxr1^{GM} complex with structures of Prp43 complexes[6-13] (teal), human Prp43 homolog DHX15 and DHX15-NKRF complexes[4,14,15] (orange), helicases DHX8[16] (blue) and Prp2[17] (cyan). ADP- or CDP-bound helicase complexes adopt highly similar RecA2 conformations, irrespective of the presence of a G-patch protein.

The RecA2 domain of Prp43:Pxr1^{GM} complex adopts a conformation most similar to ADP-bound Prp43, DHX8, DHX15, or Prp2, and clearly distinct from the RecA2 conformation adopted by ATP-analog bound Prp43 or catalytically dead mutants (ASM) of Prp43. **b,** Structural comparison of the connecting loop with respect to the bound nucleotide, measured by center of mass 3D-displacement (in Å) of the RecA2 domain of Prp43:Pxr1^{GM} complex when compared to other available structures of Prp43 and other DEAH/RHA RNA helicases. The C-terminal resolved residue of Pxr1^{GM} (W136, green) binds R270 and Y272 of Prp43, which are located at the connecting loop. This loop can adopt two different conformations regarding the position of the bound nucleotide: the distal conformation observed in ADP-bound Prp43 (blue) and Prp43:Pxr1^{GM} complex (teal), and the proximal conformation observed in *Ct*Prp43 bound to an ATP-analog (orange). In the distal conformation, residue R270 extends towards the solvent, facing away from the active site. In the proximal position, the same arginine (R273 in *C. thermophilum*) faces inwards due to a displacement of the loop and binds T118 (T121 in *C. thermophilum*) at the active site. Displacement of T271 (T274 in *C. thermophilum*) towards the active site by 7 Å brings it into direct proximity of the bound nucleotide. Adoption of the proximal conformation coincides with displacement of the RecA2 domain (center of mass 3D-displacement in Å, structures with the connecting loop in proximal position shown in orange, Prp43/DHX15 structures with RecA loop in distal position shown in blue, Prp43:Pxr1^{GM} complex shown in teal). The connecting loop is observed in the proximal position in Prp43-ATP analog, Prp43 ASM, Prp2-ATP analog, DHX36 and DHX36 with ATP analog, and in MLE-ATP analog (all show in orange), while the distal conformation is found in ADP- or CDP-bound Prp43, DHX15-ADP, DHX15-NKRF, DHX15-NKRF-ADP (blue). **c,** The C-terminal domains of Prp43 and homologous helicases adopt a position along a continuum between the fully open (Prp43-ATP analog, left) and the fully closed (DHX9-ADP, right) RNA tunnel conformation, depending on the bound nucleotide, RNA, and additional factors. Center of mass 3D-displacement (in Å) with reference to the C-terminal domains of Prp43:Pxr1^{GM} complex; shown in the plot are: Prp43 complexes[7-12] (teal), DHX9[18] (rose), DHX15 complexes[4,14,15] (green), Prp2[17,19,20] (cyan). The C-terminal domain of Prp43:Pxr1^{GM} complex adopts the open conformation, structurally closest to ATP-bound Prp43 and Prp43 ASM. The C-terminal domains in ADP-bound DHX15 (green), ADP-bound Prp2 (cyan) and ATP-analog bound Prp2 with RNA (cyan) adopt very similar conformations to their Prp43 counterparts. **(a-c)** Plots were created using the script available in the Source Data file.

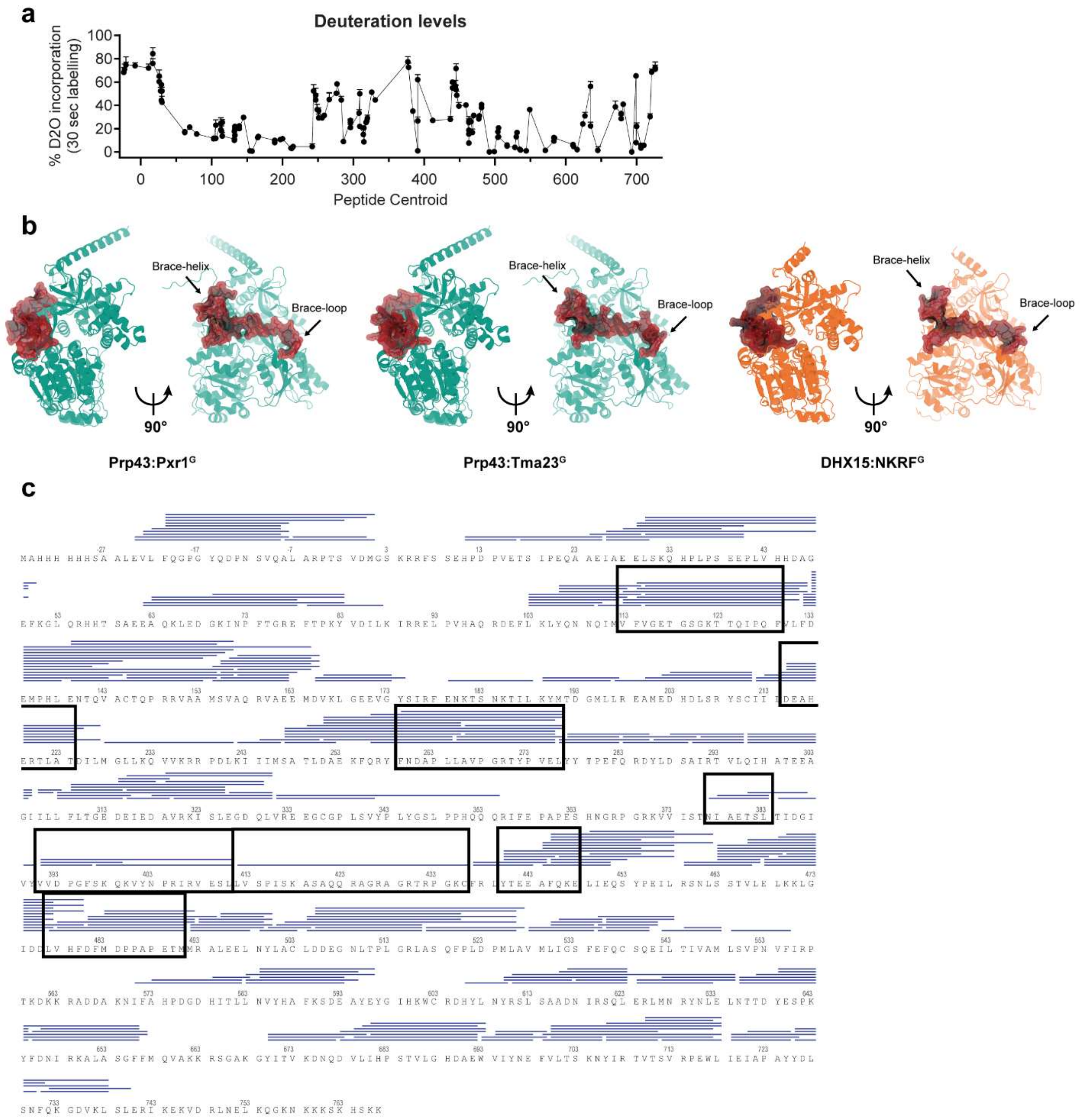

Supplementary Figure 5. HDX-MS analysis of Prp43 alone and in complex with either Pxr1^{GM} or Tma23^{GM}. a, Deuterium incorporation level of each Prp43 peptide, shown as % deuteration after a 30 sec exposure compared to a theoretical maximally deuterated value. Each dot represents one peptide, plotted based on its central residue (centroid). Values shown as percentage deuteration after 30 sec (mean +/- SD, n=3). **b,** Direct structural comparison of G-patch binding to Prp43, including the predicted AlphaFold2 models of Prp43:Pxr1^{G} and Prp43:Tma23^{G} complexes, and the experimental X-ray crystal structure of DHX15:NKRF^{G} (PDB: 6SH6)[4]. The structure of the helicase is shown as cartoon, while the model of the regulatory factor is shown as sticks overlayed with a semitransparent surface. **c**, Peptide map showing the

different H/D protected regions are indicated along the Prp43 primary sequence. Peptides shown in the uptake plots (see Fig. 7) are boxed. Residue numbering in accordance with the native protein.

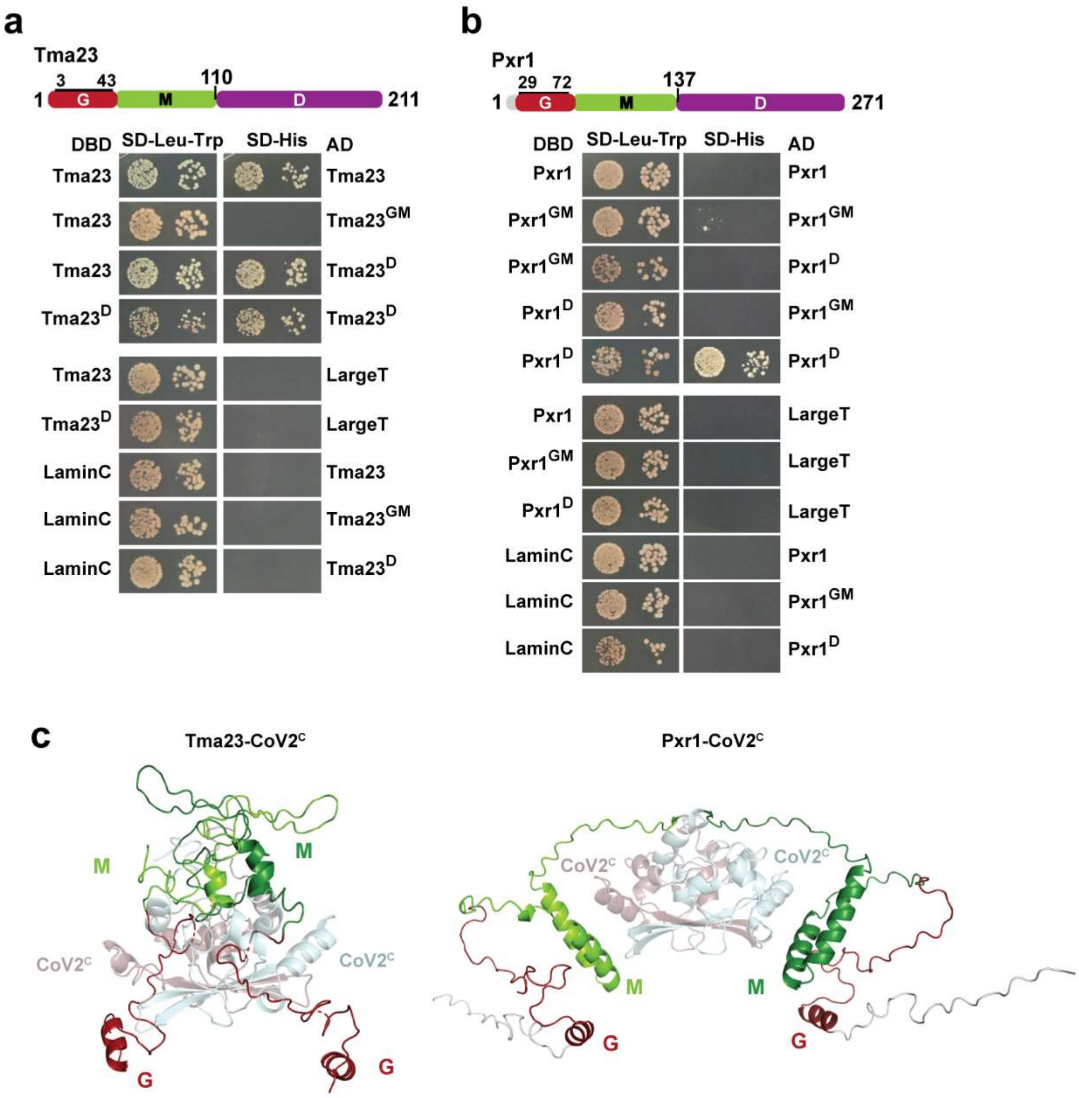

Supplementary Figure 6. Tma23^{D} and Pxr1^{D} are dimerization segments. (a-b) Plasmids encoding for wild type Tma23, Pxr1, Tma23^{GM}, Pxr1^{GM}, Tma23^{D}, or Pxr1^{D} fused to *LexA* DBD and *GAL4* AD were transformed in NMY32 strain. Transformants were spotted in 10-fold serial dilutions on the indicated selective medium and incubated at 30ºC for 2 – 4 days. LaminC and LargeT were used as negative controls. **c,** AlphaFold2 structure prediction of Tma23 or Pxr1 CoV2 chimeric constructs, shown in cartoon representation.

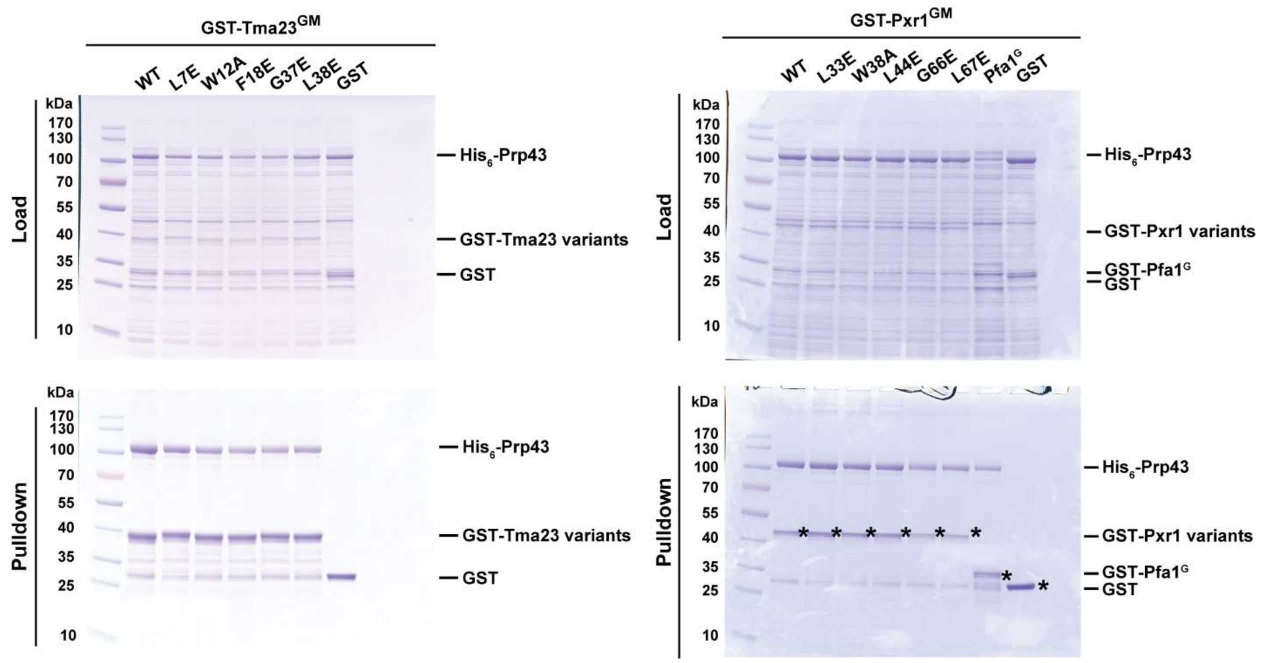

Uncropped SDS-PAGE gels: Supplementary Fig. 2b.

Supplementary References

1. Altvater, M. et al. Targeted proteomics reveals compositional dynamics of 60S pre-ribosomes after nuclear export. *Mol Syst Biol* **8**, 628 (2012).
2. Altvater, M., Schütz, S., Chang, Y. & Panse, V.G. Dissecting ribosome assembly and transport in budding yeast. *Methods Cell Biol* **122**, 437-61 (2014).
3. Demmel, L. et al. The clathrin adaptor Gga2p is a phosphatidylinositol 4-phosphate effector at the Golgi exit. *Mol Biol Cell* **19**, 1991-2002 (2008).
4. Studer, M.K., Ivanović, L., Weber, M.E., Marti, S. & Jonas, S. Structural basis for DEAH-helicase activation by G-patch proteins. *Proc Natl Acad Sci U S A* **117**, 7159-7170 (2020).
5. Pettersen, E.F. et al. UCSF ChimeraX: Structure visualization for researchers, educators, and developers. *Protein Sci* **30**, 70-82 (2021).
6. Wan, R., Yan, C., Bai, R., Lei, J. & Shi, Y. Structure of an Intron Lariat Spliceosome from Saccharomyces cerevisiae. *Cell* **171**, 120-132.e12 (2017).
7. Walbott, H. et al. Prp43p contains a processive helicase structural architecture with a specific regulatory domain. *Embo j* **29**, 2194-204 (2010).
8. He, Y., Andersen, G.R. & Nielsen, K.H. Structural basis for the function of DEAH helicases. *EMBO Rep* **11**, 180-6 (2010).
9. Robert-Paganin, J. et al. Functional link between DEAH/RHA helicase Prp43 activation and ATP base binding. *Nucleic Acids Res* **45**, 1539-1552 (2017).
10. Tauchert, M.J., Fourmann, J.B., Christian, H., Lührmann, R. & Ficner, R. Structural and functional analysis of the RNA helicase Prp43 from the thermophilic eukaryote Chaetomium thermophilum. *Acta Crystallogr F Struct Biol Commun* **72**, 112-20 (2016).
11. Tauchert, M.J., Fourmann, J.B., Lührmann, R. & Ficner, R. Structural insights into the mechanism of the DEAH-box RNA helicase Prp43. *Elife* **6**(2017).
12. Hamann, F., Enders, M. & Ficner, R. Structural basis for RNA translocation by DEAH-box ATPases. *Nucleic Acids Res* **47**, 4349-4362 (2019).
13. He, Y., Staley, J.P., Andersen, G.R. & Nielsen, K.H. Structure of the DEAH/RHA ATPase Prp43p bound to RNA implicates a pair of hairpins and motif Va in translocation along RNA. *Rna* **23**, 1110-1124 (2017).
14. Zhang, J. et al. DHX15 is involved in SUGP1-mediated RNA missplicing by mutant SF3B1 in cancer. *Proc Natl Acad Sci U S A* **119**, e2216712119 (2022).
15. Murakami, K., Nakano, K., Shimizu, T. & Ohto, U. The crystal structure of human DEAH-box RNA helicase 15 reveals a domain organization of the mammalian DEAH/RHA family. *Acta Crystallogr F Struct Biol Commun* **73**, 347-355 (2017).
16. Felisberto-Rodrigues, C. et al. Structural and functional characterisation of human RNA helicase DHX8 provides insights into the mechanism of RNA-stimulated ADP release. *Biochem J* **476**, 2521-2543 (2019).
17. Schmitt, A., Hamann, F., Neumann, P. & Ficner, R. Crystal structure of the spliceosomal DEAH-box ATPase Prp2. *Acta Crystallogr D Struct Biol* **74**, 643-654 (2018).
18. Gotur, D. et al. Development of assays to support identification and characterization of modulators of DExH-box helicase DHX9. *SLAS Discov* (2023).
19. Bai, R. et al. Mechanism of spliceosome remodeling by the ATPase/helicase Prp2 and its coactivator Spp2. *Science* **371**(2021).
20. Hamann, F. et al. The structure of Prp2 bound to RNA and ADP-BeF(3)(-) reveals structural features important for RNA unwinding by DEAH-box ATPases. *Acta Crystallogr D Struct Biol* **77**, 496-509 (2021).
