# Supplementary material for: An inhibitory segment within G-patch activators tunes Prp43-ATPase activity during ribosome assembly
Source: Nat Commun. 2024 Nov 22;15:10150. doi: 10.1038/s41467-024-54584-5 (PMC11584650; doi:10.1038/s41467-024-54584-5)
Supplement: Supplementary file 2 — Description of Additional Supplementary Files [file 41467_2024_54584_MOESM2_ESM.pdf]

Description of Additional Supplementary Files

File name: Supplementary Data 1
Description: HDX-MS studies of Prp43 alone and in complex with $Tma23^{GM}$ and $Pxr1^{GM}$.

File name: Supplementary Data 2
Description: XL-MS analysis of Prp43:$Tma23^{GM}$ and Prp43:$Pxr1^{GM}$ complexes.
