# Supplementary material for: An inhibitory segment within G-patch activators tunes Prp43-ATPase activity during ribosome assembly
Source: Nat Commun. 2024 Nov 22;15:10150. doi: 10.1038/s41467-024-54584-5 (PMC11584650; doi:10.1038/s41467-024-54584-5)
Supplement: Supplementary file 5 — Reporting Summary [file 41467_2024_54584_MOESM5_ESM.pdf]

nature portfolio

Corresponding author(s): Vikram Govind Panse

Last updated by author(s): Oct 17, 2024

Reporting Summary

Nature Portfolio wishes to improve the reproducibility of the work that we publish. This form provides structure for consistency and transparency in reporting. For further information on Nature Portfolio policies, see our Editorial Policies and the Editorial Policy Checklist.

Statistics

For all statistical analyses, confirm that the following items are present in the figure legend, table legend, main text, or Methods section.

n/a	Confirmed	
☐	☒	The exact sample size (*n*) for each experimental group/condition, given as a discrete number and unit of measurement
☐	☒	A statement on whether measurements were taken from distinct samples or whether the same sample was measured repeatedly
☐	☒	The statistical test(s) used AND whether they are one- or two-sided<br>*Only common tests should be described solely by name; describe more complex techniques in the Methods section.*
☒	☐	A description of all covariates tested
☒	☐	A description of any assumptions or corrections, such as tests of normality and adjustment for multiple comparisons
☐	☒	A full description of the statistical parameters including central tendency (e.g. means) or other basic estimates (e.g. regression coefficient) AND variation (e.g. standard deviation) or associated estimates of uncertainty (e.g. confidence intervals)
☐	☒	For null hypothesis testing, the test statistic (e.g. *F*, *t*, *r*) with confidence intervals, effect sizes, degrees of freedom and *P* value noted<br>*Give P values as exact values whenever suitable.*
☒	☐	For Bayesian analysis, information on the choice of priors and Markov chain Monte Carlo settings
☒	☐	For hierarchical and complex designs, identification of the appropriate level for tests and full reporting of outcomes
☒	☐	Estimates of effect sizes (e.g. Cohen's *d*, Pearson's *r*), indicating how they were calculated

Our web collection on statistics for biologists contains articles on many of the points above.

Software and code

Policy information about availability of computer code

Data collection	Details are described in Methods and Supplementary Information in Sections: Tandem affinity purification, Sample preparation for LC-MS/MS, LC-MS analysis, Protein identification and label-free quantification, Hydrogen-Deuterium Exchange mass spectrometry.<br>Fluorescence microscopy: Images were obtained by Leica Application Suite X software.<br>Cryo-EM models built with Chimera (v1.15)<br>Cryo-EM: Serial 3.7.11
Data analysis	Microscopy images were acquired by Leica Application Suite X software and processed using ImageJ (v1.50e).<br>Mass Spectometry of Prp43-TAP: FragPipe (version 16.0), MSFragger (version 3.3), Philosopher (version 4.4.0), IonQuant 1.7.17, MSFragger search engine 3.4.<br>Figures of molecular structures were created by PyMOL (v2.3.3)<br>Sequence alignment: generated by MAFFT software (v7) and visualized by Jalview (v2.11.2.5)<br>Protein structural modelling: AlphaFold2 (v2.0.1)<br>Cryo-EM: MotionCorr2, GCTF, Relion 3.1 beta, PHENIX v1.19.1; Coot v0.8.9.2; Pymol v2.3.5; Proteome Discoverer 2.4

For manuscripts utilizing custom algorithms or software that are central to the research but not yet described in published literature, software must be made available to editors and reviewers. We strongly encourage code deposition in a community repository (e.g. GitHub). See the Nature Portfolio guidelines for submitting code & software for further information.

April 2023

Data

Policy information about availability of data

All manuscripts must include a data availability statement. This statement should provide the following information, where applicable:

- Accession codes, unique identifiers, or web links for publicly available datasets
- A description of any restrictions on data availability
- For clinical datasets or third party data, please ensure that the statement adheres to our policy

The mass spectrometry dataset of Prp43-TAP generated in this study has been deposited in the ProteomeXchange Consortium via the PRIDE partner repository under accession code PXD048382.

The XL-MS data are available in the PRIDE repository under accession code PXD047173.

The HDX-MS data generated in this study are deposited in the ProteomeXchange Consortium via the PRIDE partner repository, under accession code PXD046892.

The Cryo-EM density map was deposited in the EMDB database, under accession code EMD-19078. The refined atomic model was submitted to the PDB database, with accession code PDB 8RDY.

Research involving human participants, their data, or biological material

Policy information about studies with human participants or human data. See also policy information about sex, gender (identity/presentation), and sexual orientation and race, ethnicity and racism.

Reporting on sex and gender	Not applicable
Reporting on race, ethnicity, or other socially relevant groupings	Not applicable
Population characteristics	Not applicable
Recruitment	Not applicable
Ethics oversight	Not applicable

Note that full information on the approval of the study protocol must also be provided in the manuscript.

Field-specific reporting

Please select the one below that is the best fit for your research. If you are not sure, read the appropriate sections before making your selection.

☒ Life sciences ☐ Behavioural & social sciences ☐ Ecological, evolutionary & environmental sciences

For a reference copy of the document with all sections, see nature.com/documents/nr-reporting-summary-flat.pdf

Life sciences study design

All studies must disclose on these points even when the disclosure is negative.

Sample size	Samples for label-free quantitative mass spectrometry were prepared, processed and acquired as 4 biological replicates. All biochemistry and cell-biological studies were performed with defined components (Tma23/Pxr1/Pfa1/Prp43), hence in these instances sample size is not applicable. Cryo-EM: the number of particles chosen for cryo-EM reconstructions are similar or exceed the data sizes of similar studies. No mathematical sample size calculation was performed. Detailed information is provided in the cryo-EM workflow presented in the Supplementary information.
Data exclusions	All the mass spectrometry, biochemical and cell-biological analyses are presented. No data was excluded. All genetic experiments were performed at least three times, no data was excluded. All biochemical purifications for cryo-EM, XL-MS, and HDX-MS were performed at least 3 times with comparable results.
Replication	All ATPase assays, biochemical binding assays, genetic and cell-biological experiments were performed at least 3 times; where applicable appropriate errors are provived and stated in the Figure legend/text.
Randomization	No randomization was performed.
Blinding	All mass spectrometry biochemical and biophysical employ defined components (Tma23/Pxr1/Pfa1/Prp43) and cell-biological studies employed defined yeast mutant cells expressing GFP fusions, hence binding is not relevant.

Reporting for specific materials, systems and methods

We require information from authors about some types of materials, experimental systems and methods used in many studies. Here, indicate whether each material, system or method listed is relevant to your study. If you are not sure if a list item applies to your research, read the appropriate section before selecting a response.

Materials & experimental systems

n/a	Involved in the study
☐	☒ Antibodies
☒	☐ Eukaryotic cell lines
☒	☐ Palaeontology and archaeology
☒	☐ Animals and other organisms
☒	☐ Clinical data
☒	☐ Dual use research of concern
☒	☐ Plants

Methods

n/a	Involved in the study
☒	☐ ChIP-seq
☒	☐ Flow cytometry
☒	☐ MRI-based neuroimaging

Antibodies

Antibodies used

Primary antibodies were used in the following dilutions: α-Prp43 (1:500, this study); α-Tma23 (1:1000, this study): α-Pxr1 (1:1000, this study). α-CBP (1:1000, Cat. No. 07-482, Merck AG). The secondary HRP-conjugated α-rabbit antibody (1:1000, Cat. No. I5006; Merck AG).

Validation

Primary polyclonal antibodies against Tma23 and Pxr1 were raised in rabbits using the following peptide sequences: DGEAWWERLFDGHLKNLDV and KQKRAALMDSKALNEIFM, respectively. All antibodies were validated using whole cell yeast lysates and recombinant proteins.

Plants

Seed stocks

No plants used in the study

Novel plant genotypes

No plants used in the study

Authentication

No plants used in the study
